# The Developmental Context of Social Hopefulness and Suicide Risk

**DOI:** 10.1093/geroni/igaa057.1030

**Published:** 2020-12-16

**Authors:** Ali Molaie, Adrienne Chong, Jane Fisher

**Affiliations:** University of Nevada, Reno, Reno, Nevada, United States

## Abstract

Social disconnectedness, or thwarted belongingness (TB) has been documented as an important risk factor for suicide across the lifespan. Perceived time left (PTL) may impact perceptions of hopelessness for social connectedness and therefore inform developmentally sensitive trajectories of suicide risk. This study examined interpersonal conditions associated with social hopelessness in younger and older adults in order to identify variables important for conceptualizing suicidality across the lifespan. We compared younger and older adults’ perceptions of social hopelessness for characters in vignettes that were depicted as having high or low TB (i.e., described as lonely or not lonely) and high or low PTL (i.e., described as being 35-years-old or 85-years-old). Additionally, we examined the relation between social hopelessness and suicide risk (using the Suicide Behaviors Questionnaire—Revised). Participants included 135 younger (M = 19.32) and 69 older (M = 74.91) adults. Older adults endorsed less social hopelessness than did younger adults on the High TB/Low PTL vignette, t(102.15) = -4.88, p < 0.001, as well as the Low TB/Low PTL vignette, t(194) = -2.04, p = 0.04. Participants with higher suicide risk also endorsed higher social hopelessness on the High TB/Low PTL vignette than did participants with lower suicide risk, t(194) = -2.10, p = 0.04. Younger adults and participants with higher suicide risk across both age groups reported less optimism for characters’ future social connectedness, particularly for those portrayed as older. This study provides support for the importance of developmentally informed conceptualizations of risk factors for suicide, including social hopelessness.

